# Supplementary material for: Characterization of the RAD52 Gene in the Budding Yeast Naumovozyma castellii
Source: Genes (Basel). 2023 Oct 5;14(10):1908. doi: 10.3390/genes14101908 (PMC10606518; doi:10.3390/genes14101908)
Supplement: Supplementary file 1 [file genes-14-01908-s001.zip › genes-2638851-supplementary.pdf]

Supplementary Material

Supplementary Figures:

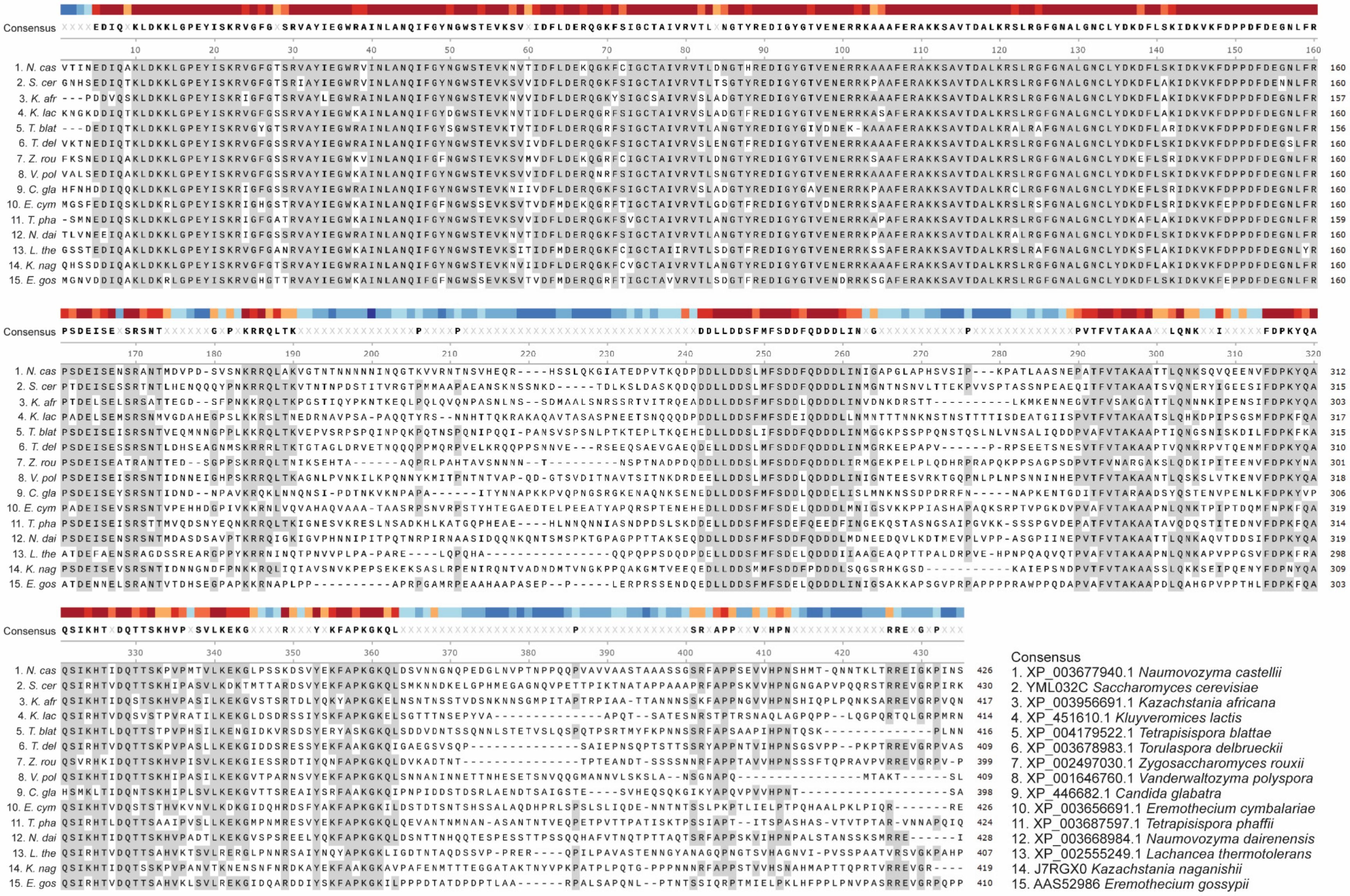
Consensus
1. XP_003677940.1 Naumovozyma castellii
2. YML032C Saccharomyces cerevisiae
3. XP_003956691.1 Kazachstania africana
4. XP_451610.1 Kluyveromices lactis
5. XP_004179522.1 Tetrapisispora blattae
6. XP_003678983.1 Torulaspora delbrueckii
7. XP_002497030.1 Zygosaccharomyces rouxii
8. XP_001646760.1 Vanderwaltozyma polyspora
9. XP_446682.1 Candida glabatra
10. XP_003656691.1 Eremothecium cymbalariae
11. XP_003687597.1 Tetrapisispora phaffii
12. XP_003668984.1 Naumovozyma dairenensis
13. XP_002555249.1 Lachancea thermotolerans
14. J7RGX0 Kazachstania naganishii
15. AAS52986 Eremothecium gossypii

Supplementary Figure S1. Multiple sequence alignment of Rad52 homologs in the Saccharomycetaceae family. The primary amino acid structure of Rad52 orthologs from the Saccharomycetaceae family was retrieved from the eggNOG database. Before alignment the sequences were cleaved to contain only the regions that matched the “DNA repair and recombination protein RAD52, RAD59” classification described in PANTHER (PTHR12132) with HMMER. Multiple sequence alignment was performed by MUSCLE and the results of the alignment were illustrated using SnapGene. Red boxes represent conserved amino acid residues and blue boxes represent less conserved ones. Residues colored grey in the sequence alignment represent identical amino acids to the consensus sequence.

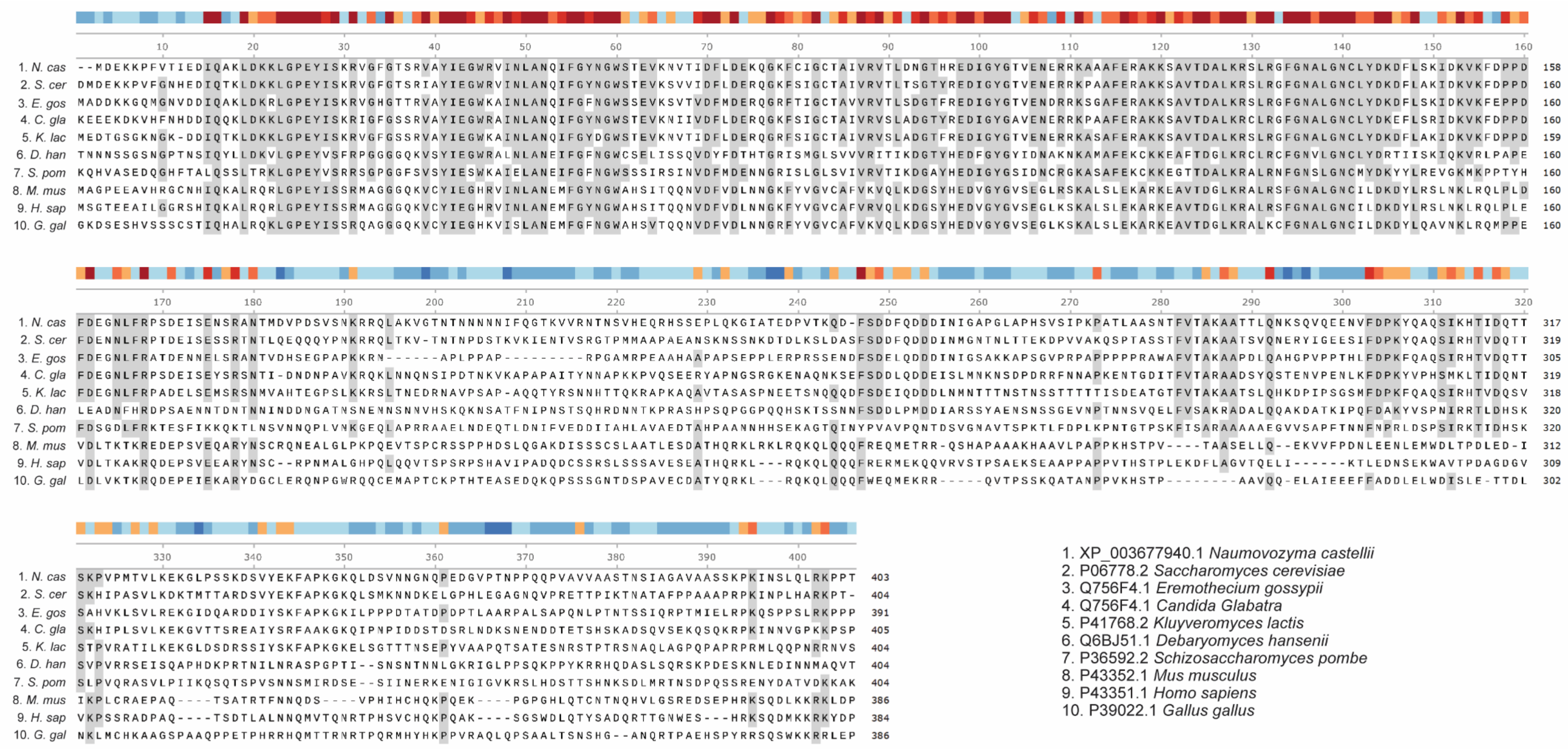
1. XP_003677940.1 Naumovozyma castellii
2. P06778.2 Saccharomyces cerevisiae
3. Q756F4.1 Eremothecium gossypii
4. Q756F4.1 Candida Glabatra
5. P41768.2 Kluyveromyces lactis
6. Q6BJ51.1 Debaryomyces hansenii
7. P36592.2 Schizosaccharomyces pombe
8. P43352.1 Mus musculus
9. P43351.1 Homo sapiens
10. P39022.1 Gallus gallus

Supplementary Figure S2. Multiple sequence alignment of proteins identified by BLAST. BLAST against the SWISS-PROT database using *N. castellii* RAD52 amino acid sequence as query was performed locally. The proteins listed by accession number and name of the species at the bottom left of the figure, include the proteins selected by filtering the BLAST results for Rad52 homologs in order of increasing E-values. The primary structure of the proteins was aligned using MUSCLE and the results of the alignment were illustrated using SnapGene. Red boxes represent conserved amino acid residues and blue boxes represent less conserved ones. Residues colored grey in the sequence alignment represent identical amino acids to the *N. castellii* sequence.

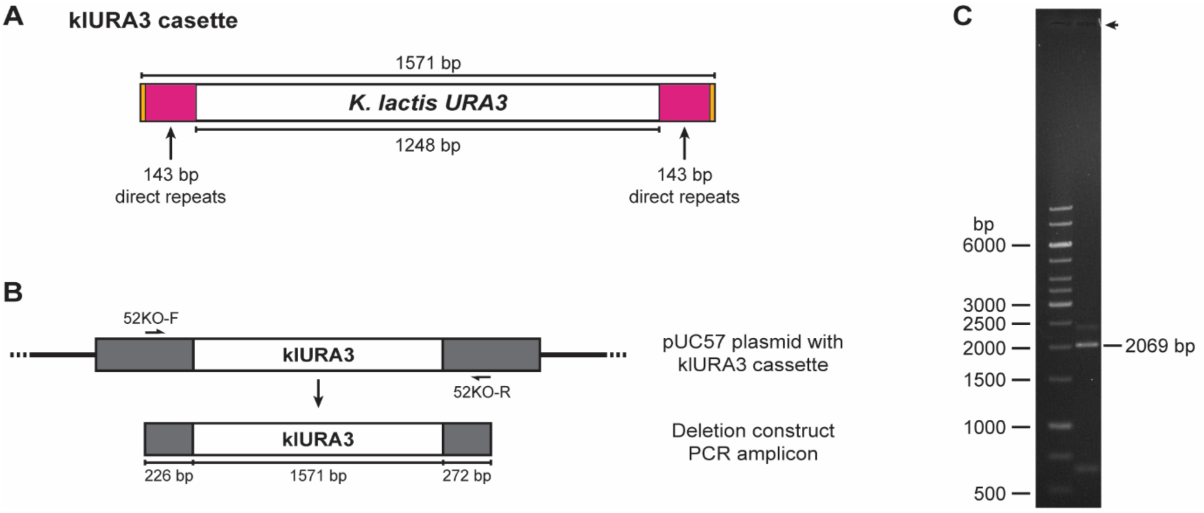

Supplementary Figure S3. The *Kluyveromyces lactis URA3* cassette (klURA3) was used to replace the *RAD52* gene in *N. castellii*. A) Schematic of the klURA3 cassette. The klURA3 cassette contains the *K. lactis URA3* marker gene flanked by short direct repeats of 143 bp (pink) and small remnants of the pWJ1042 donor plasmid (yellow, 18 and 19 bp at each end). The direct repeats will allow the excision of the marker gene through recombination by circularization of the DNA and recombination between these homologous regions. B) Schematic depicting the creation of the *RAD52* deletion construct. The pUC57 plasmid containing the klURA3 cassette flanked by the upstream and downstream sequences of the *N. castellii RAD52* locus was bioinformatically designed by the authors and manufactured by the GenScript company. To create the *RAD52* deletion construct the klURA3 cassette was amplified by PCR utilizing primers that bind to the *RAD52* flanking region, 226 and 272 bp upstream and downstream, respectively. After PCR amplification, the *RAD52* deletion construct has a length of 2069 bp. C) Confirmation of the PCR amplification of the *RAD52* deletion construct. The purified PCR product was resolved in a 0.8% agarose gel in 0.5x TBE.

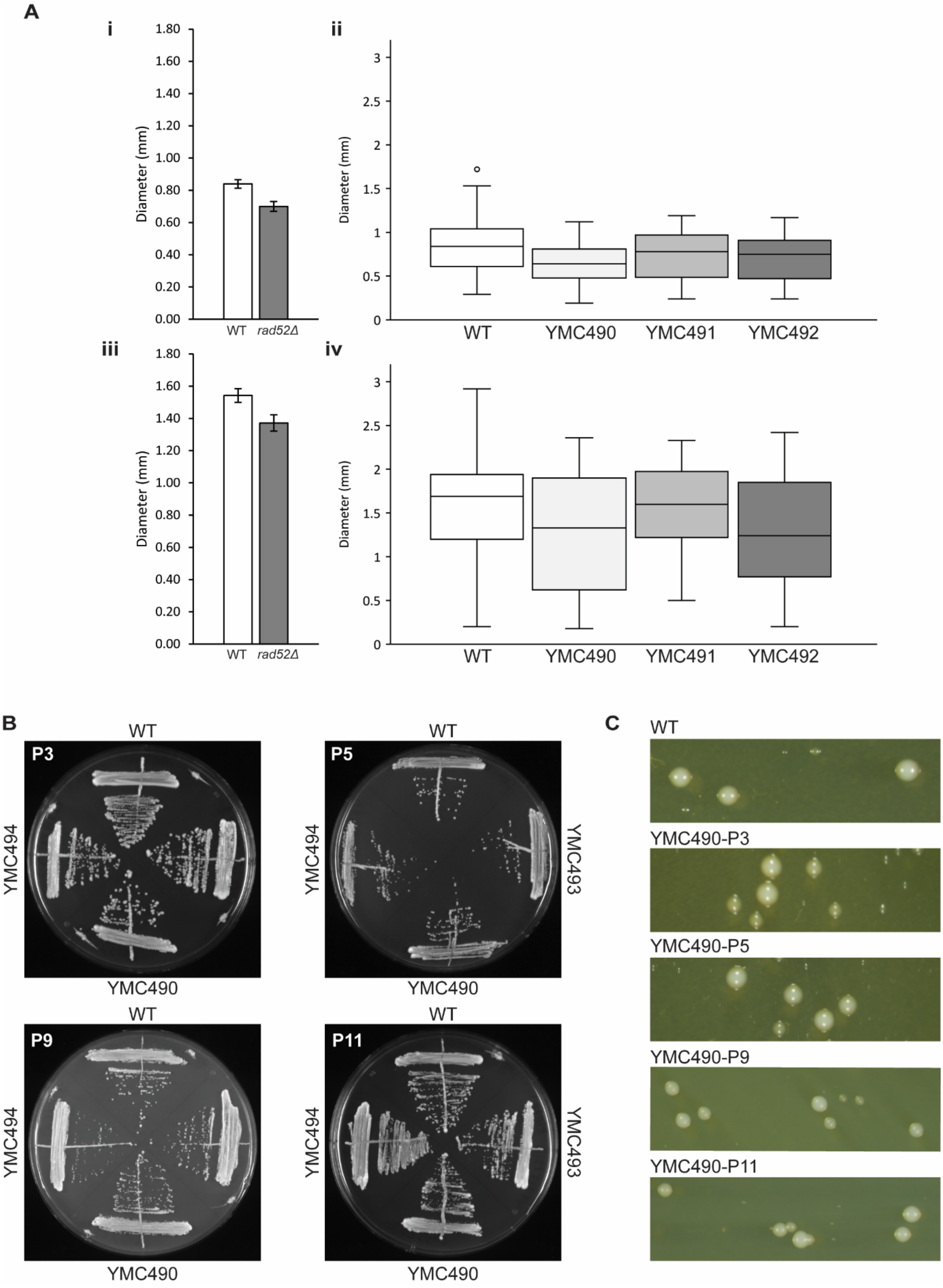

Supplementary Figure S4. ***N. castellii rad52Δ*** **mutants have WT colony morphology and growth on solid media, but somewhat smaller colony size.** A) Colony size estimation for WT (YMC48) and *rad52Δ* mutant strains. Colony measurements were taken after 2 (i-ii) or 3 days (iii-iv) of growth at 25°C. The average results are presented in a histogram, together with a boxplot to visualize the distribution of sizes. The average size of colonies was calculated for: i) WT (n=129) and *rad52Δ*

(n=239) and iii) WT (n=181) and *rad52Δ* (n=424). Bars represent SEM. B-C) Mutant strains can sustain long-term growth in solid media. The wild-type colony morphology is maintained after several generations of growth. The mutant *rad52Δ* strains YMC490, YMC493 and YMC494, together with the WT strain YMC48, were passaged multiple times on YPD plates and photographed at the different passages indicated (P3, P5, P9 and P11). C) Close-up photographs of the YMC490 colonies on the plate.

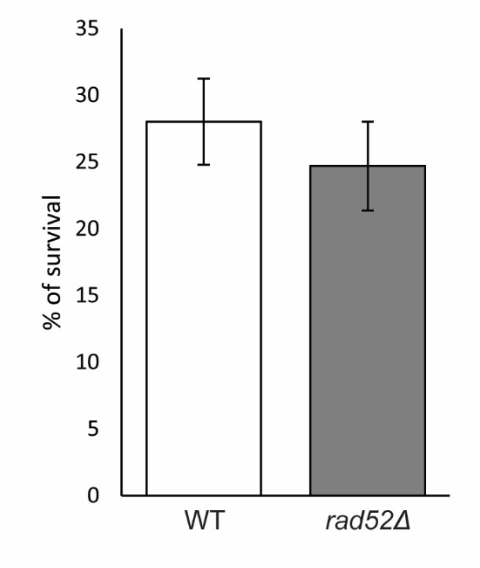

Supplementary Figure S5. ***N. castellii rad52Δ*** **cells remain viable.** Comparison of cell viability between WT (YMC48) and *rad52Δ* mutants. The percentage of survival was estimated as the ratio of the number of colony forming units and the total number of cells plated. Cells were spread on YPD solid media and incubated at 25°C for 3 days. The bars represent SEM for biological replicates for both WT (n=12) and *rad52Δ* (n=34). No significant difference was found between the mean values (Two-sided Student's t-test, p=0.384).

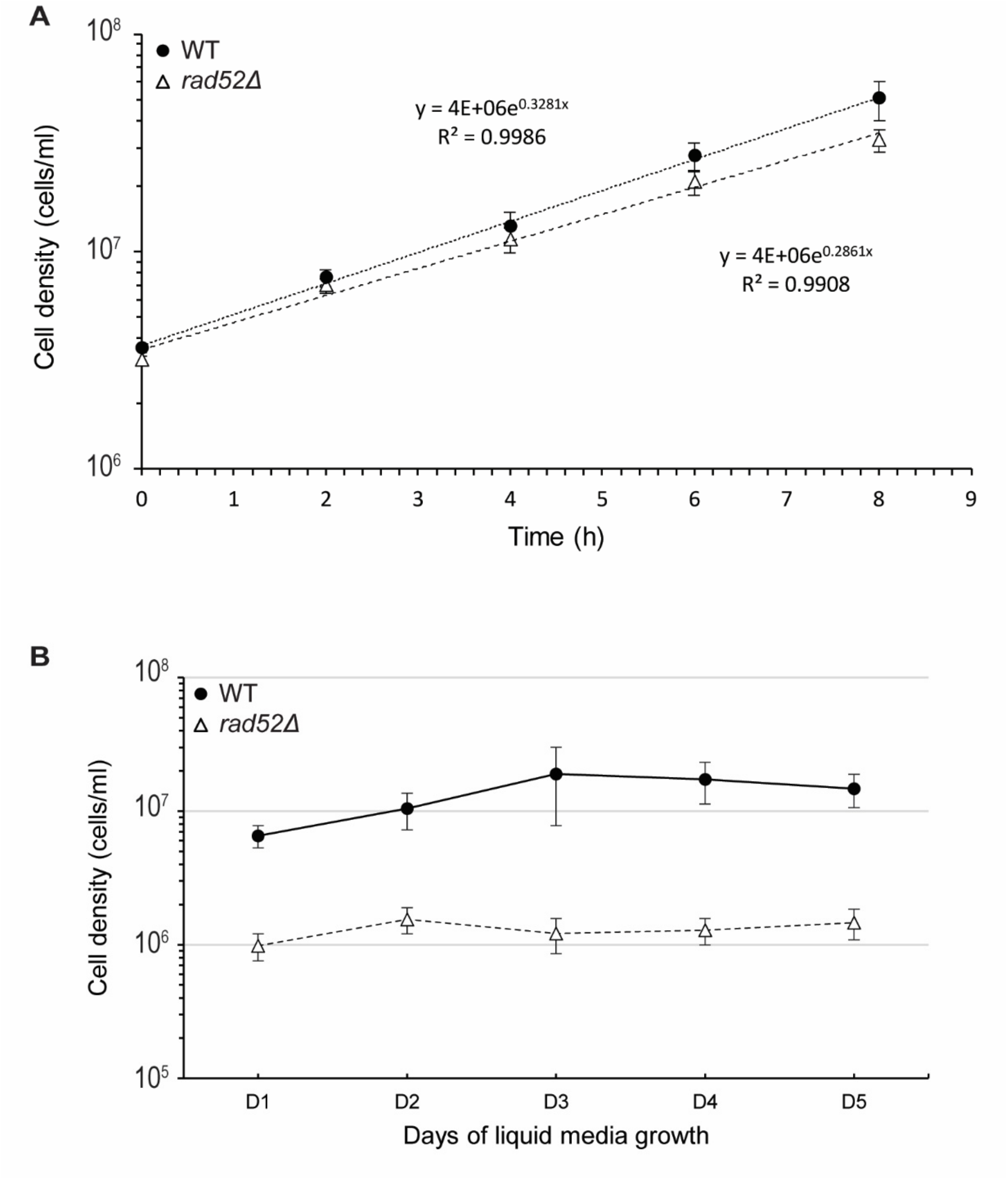

Supplementary Figure S6. *N. castellii rad52Δ* mutants have decreased growth rate compared to WT. A) Determination of the doubling time. The cell densities obtained during the 8 hours growth analysis (Figure 3D) were plotted and used to calculate a trendline that best fits the measurements. The formula for the exponential trendline was utilized to determine the doubling time of the WT (R^2=0.9986) and *rad52Δ* (R^2=0.9908) strains. B) Long-term growth analysis in liquid media was performed by starting a 10 ml YPD culture at a cell density of $1x10^3$ cells/ml from an overnight starter culture. After 24 hours of growth at 25 °C, the cell density of the culture was measured (D1). A new culture was started at a density of $1x10^3$ cells/ml as described previously using the previous culture as a starter culture, and this process was repeated for five days. The average cell density values of 3 biological replicates are represented in the graph.

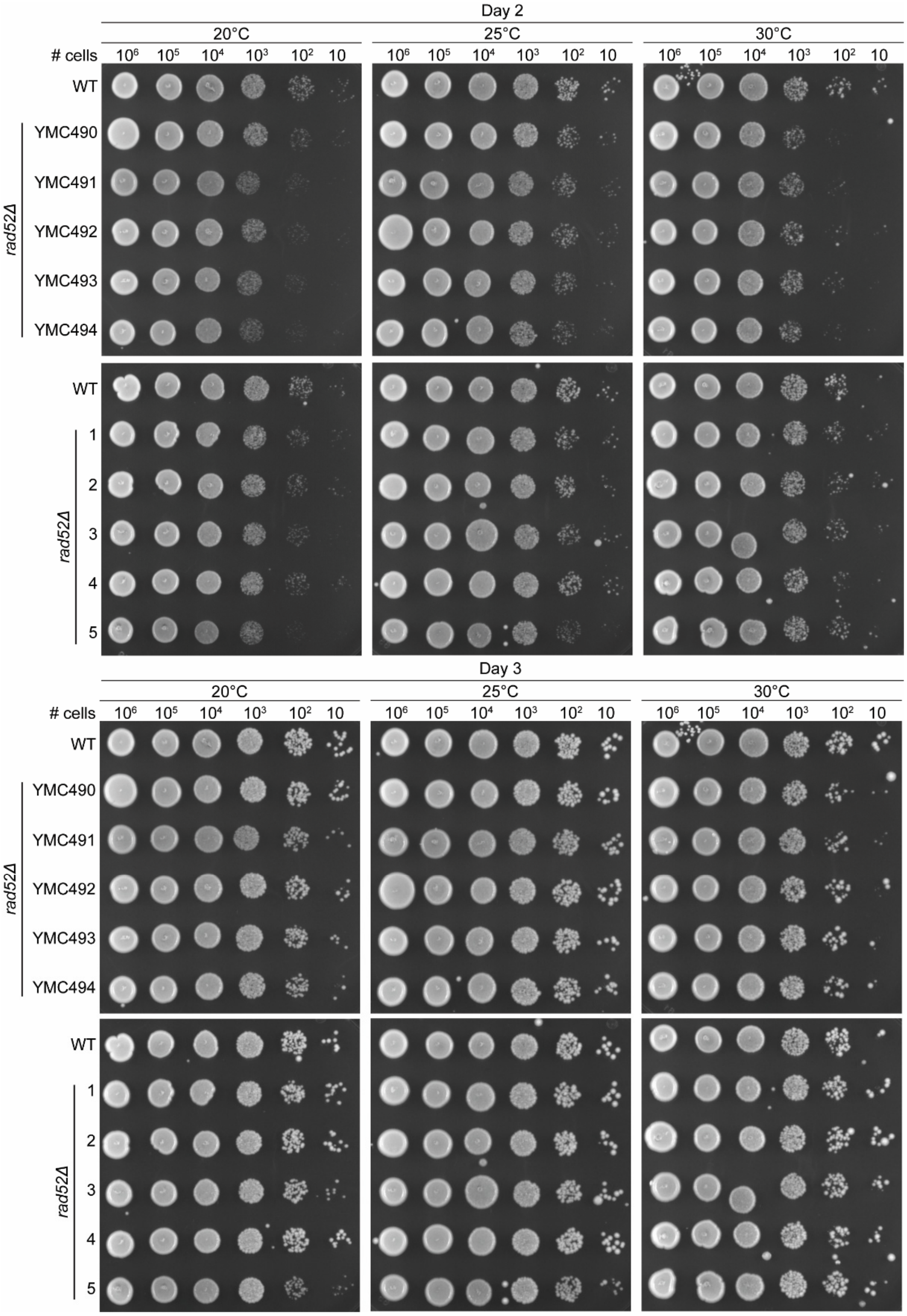

Supplementary Figure S7. Temperature sensitivity spot assay. Tenfold serial dilutions of WT and *rad52Δ* strains were spotted on YPD plates. In total 10 strains were plated in parallel, 5 of them were unnamed and labelled 1-5. Cells were grown for 2-3 days at 20, 25 and 30 °C as indicated.

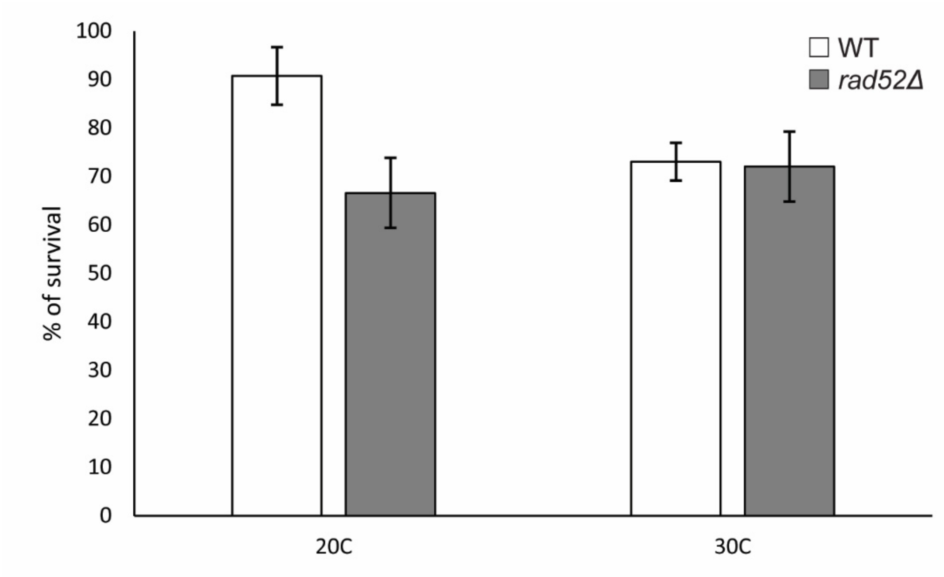

Supplementary Figure S8. Similar to WT, the *rad52Δ* mutants show slightly lower growth at sub-optimal temperatures. Viability assay was performed at the optimal growth temperature of 25°C as well as sub-optimal temperatures of 20°C and 30°C, for WT and *rad52Δ* mutant strains. The percentage of survival was estimated based on the number of colony forming units obtained in YPD solid media after incubation at 20 and 30 °C for 3 days normalized to the colonies obtained at 25°C for the respective strain. The bars represent SEM for n=3 and n=9 biological replicates for WT and *rad52Δ* respectively. Two-sided Student's t-test revealed a significant difference between the mean viability values of WT and *rad52Δ* at 20 °C ($p<0.05$) and none for the values at 30 °C ($p=0.99$).

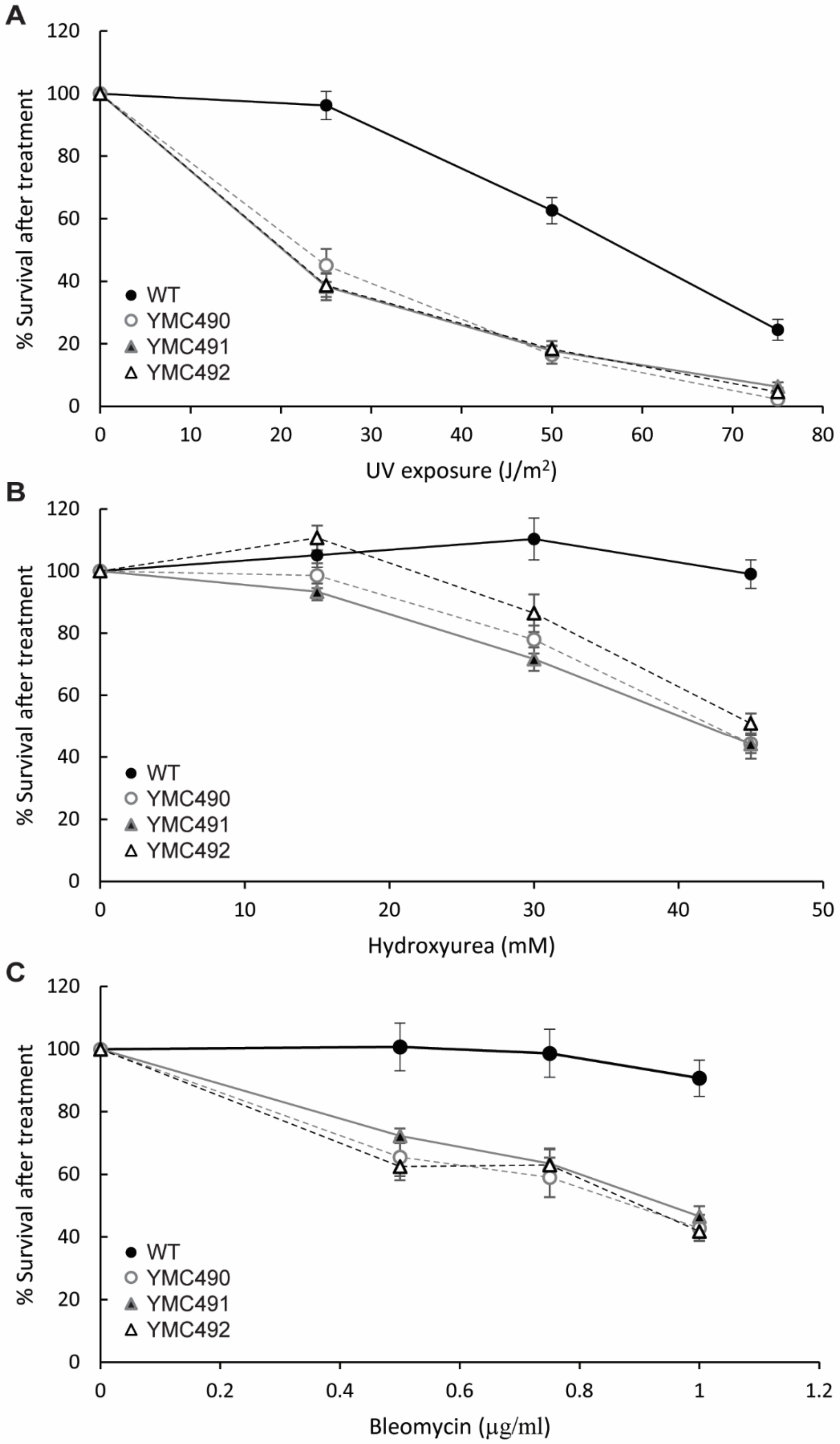

Supplementary Figure S9. Quantitative survival curves of *rad52Δ* strains treated with genotoxic agents. The data in Figure 5, is represented as individual plots of the *rad52Δ* strains. Cells from WT and 3 different *rad52Δ* strains (YMC490, YMC491 and YMC492) were spread onto YPD solid media and exposed to different doses of UV irradiation, or YPD solid media containing hydroxyurea or bleomycin at different concentrations. After incubation for 3 days at 25 °C, the number of colony forming units were counted for each treatment and normalized to the non-treated control plate. Bars represent SEM for n=3.
